# Complete chloroplast genome of *Hordeum brevisubulatum*: Genome organization, synonymous codon usage, phylogenetic relationships, and comparative structure analysis

**DOI:** 10.1371/journal.pone.0261196

**Published:** 2021-12-13

**Authors:** Guangxin Cui, Chunmei Wang, Xiaoxing Wei, Hongbo Wang, Xiaoli Wang, Xinqiang Zhu, JinHua Li, Hongshan Yang, Huirong Duan

**Affiliations:** 1 Lanzhou Institute of Husbandry and Pharmaceutical Science, Chinese Academy of Agricultural Sciences, Lanzhou, Gansu, China; 2 Academy of Animal and Veterinary Sciences, Qinghai University, Xining, Qinghai, China; 3 Laboratory of Quality & Safety Risk Assessment for Livestock Products, Ministry of Agriculture and Rural Affairs, Lanzhou, Gansu, China; Saint Mary’s University, CANADA

## Abstract

**Background:**

*Hordeum brevisubulatum*, known as fine perennial forage, is used for soil salinity improvement in northern China. Chloroplast (cp) genome is an ideal model for assessing its genome evolution and the phylogenetic relationships. We *de novo* sequenced and analyzed the cp genome of *H*. *brevisubulatum*, providing a fundamental reference for further studies in genetics and molecular breeding.

**Results:**

The cp genome of *H*. *brevisubulatum* was 137,155 bp in length with a typical quadripartite structure. A total of 130 functional genes were annotated and the gene of *accD* was lost in the process of evolution. Among all the annotated genes, 16 different genes harbored introns and the genes of *ycf3* and *rps12* contained two introns. Parity rule 2 (PR2) plot analysis showed that majority of genes had a bias toward T over A in the coding strand in all five *Hordeum* species, and a slight G over C in the other four *Hordeum* species except for *H*. *bogdanil*. Additionally, 52 dispersed repeat sequences and 182 simple sequence repeats were identified. Moreover, some unique SSRs of each species could be used as molecular markers for further study. Compared to the other four *Hordeum* species, *H*. *brevisubulatum* was most closely related to *H*. *bogdanii* and its cp genome was relatively conserved. Moreover, inverted repeat regions (IRa and IRb) were less divergent than other parts and coding regions were relatively conserved compared to non-coding regions. Main divergence was presented at the SSC/IR border.

**Conclusions:**

This research comprehensively describes the architecture of the *H*. *brevisubulatum* cp genome and improves our understanding of its cp biology and genetic diversity, which will facilitate biological discoveries and cp genome engineering.

## Introduction

Soil salinity is a serious threat to plant growth. Consequently, the breeding of salt-tolerant plants plays a vital role in the exploitation and utilization of saline land [1]. *Hordeum brevisubulatum* (Trin.) Link, or wild barley, is widely distributed in northern China and is known for its use as fine perennial forage. The plant is characterized by its adaptability, good palatability for livestock, and high regeneration capacity. Wild barley can also be used ecologically for soil improvement since it is one of the most salt-tolerant Triticeae species [2]. *H*. *brevisubulatum* has been domesticated for nearly 50 years in China. Since 1976, *H*. *brevisubulatum* has been promoted in Jilin Province because of its higher biomass and seed yield than *H*. *bogdanii* [3, 4]. The first cultivar of *H*. *brevisubulatum*, *‘*Junxu No.1*’*, was approved in China in 2003 [5]. However, it took nearly 10 years to breed this variety by conventional breeding methods, which included chemical mutagenesis, fine individual selection and cultivation, variety comparison testing, regional testing, and promotion testing. The use of conventional breeding to create new cultivars generally requires at least 10 years. In contrast, molecular breeding is economical and effective, shortens breeding time, and has been used extensively in various plants including cereals, vegetables, fruits, cash crops, and ornamental plants [6]. Molecular breeding is based on genetic information and has helped developing many new cultivars with high yield, quality, and resistance to stress. Until now, research on *H*. *brevisubulatum* has focused mainly on its biological characteristics, salt tolerance mechanisms, and disease resistance [1, 7, 8]. The lack of genetic information on *H*. *brevisubulatum* slows the pace of cultivation of different varieties. In addition, although *H*. *brevisubulatum* has been studied as a model to understand the salt-tolerance mechanisms of barley cereal crops, the genetic relationship between barley and *H*. *brevisubulatum* is still unclear. This knowledge would enable scientists to exploit *H*. *brevisubulatum* germplasm and accelerate the pace of breeding [9].

Owing to advancements in high-throughput sequencing, nearly 22,000 chloroplast (cp) genomes have been completely identified and deposited in the National Center for Biotechnology Information (NCBI) as of early April 2020. Cp is the most important plastid and semi-autonomous organelle containing independent DNA information. Among the three independent genomes with genetic information in plants, the cp genome is conservative and relatively small in size. In general, cp genomes are usually between 107 and 218 kb and encode 120–130 unique genes in a typical quadripartite cycle comprising a pair of inverted repeat regions (IRa and IRb). The regions are separated by a large single copy (LSC) region and a small single copy (SSC) region [10]. The cp genome in conifers, algae, and some legume species is rearranged repeatedly mainly due to expansion, contraction, or the loss of IR regions [10–13]. For the majority of higher plants, the cp genome is highly conservative in gene number, arrangement order, and function [10, 14], since information carried on the cp genome is inherited only from the female parent, and fewer nucleotide substitutions and genome structure rearrangements have occurred compared to the nuclear genome [15–17]. Hence, as an ideal model to unravel genome evolution and the phylogenetic relationships in complex angiosperm families [18, 19], the cp genome has been used widely in gene mapping, variety identification, plant barcode sequence screening, population genetics, gene diversity studies, and molecular assisted breeding [10, 20]. Therefore, analysis of cp genome organization in *H*. *brevisubulatum*, its phylogenetic relationship and structure comparative analysis with other *Hordeum* species would be both beneficial and interesting.

In the current study, the whole cp genome of *H*. *brevisubulatum* was constructed by initially using next-generation sequencing and applying a combination of *de novo* and reference-guided assembly. Then, the whole cp genome sequence of *H*. *brevisubulatum* was described, repeat sequences and simple sequence repeats (SSRs) were analyzed. Furthermore, an evolutionary phylogenetic tree was constructed and the cp genome structure of *H*. *brevisubulatum* was compared with another four *Hordeum* species. Results from this study could provide fundamental genetic reference for future biological research and molecular breeding programs of *H*. *brevisubulatum*.

## Materials and methods

### Plant material

Fresh leaf samples of *H*. *brevisubulatum* were collected in July 2019 from the Lanzhou Scientific Observation and Experiment Field Station of the Ministry of Agriculture for Ecological Systems in the Loess Plateau area (36°01′N, 103°45′E, and altitude 1700 m above sea level), Lanzhou Institute of Husbandry and Pharmaceutical Science, Chinese Academy of Agricultural Sciences, Gansu, China. The voucher specimen was formally identified by an expert on plant taxonomy and kept in the Herbarium of Lanzhou Institute of Husbandry and Pharmaceutical Science, Chinese Academy of Agricultural Sciences (CYSLS-HbWang20190722). Samples were frozen immediately in liquid nitrogen, conserved in drikold and delivered to Beganen Tech Solution CO., Ltd (Wuhan, China) for cp genome extraction and sequencing, and then the data were assembled and further analyzed by Genepioneer Biotechnologies Co., Ltd (Wuhan, China).

### DNA extraction, sequencing, and assembly

Genomic DNA was isolated by the Plant Genomic DNA Rapid Extraction Kit (Biomed Gene Technology) by the modified CTAB method [21]. A Qubit Fluorometer (Invitrogen) and 1% agarose gel electrophoresis were used to detect DNA integrity and quality. A library (350 bp) was constructed using pure DNA according to the manufacturer’s instructions (NEBNext^®^Ultra^TM^ DNA Library Prep Kit for Illumina^®^). The library was sequenced with an Illumina NovaSeq platform and 150 bp paired-end reads were generated. Among the Illumina PCR adapter reads, more than 5% of reads of unknown origin and low-quality reads were filtered using SOAPnuke software (version: 1.3.0). A total of 6.8 GB of clean data was yielded for *H*. *brevisubulatum*. Bowtie 2 v2.2.4 software was used to compare the cp genome database built by Genepioneer Biotechnologies Co. Ltd. and the matching clean reads were selected for subsequent assembly. The corrected sequence was arranged according to the procedure described by Sun *et al*. [22] to obtain the complete circular cp genome sequence.

### Annotation and analysis of the Cp genome sequences

Three methods were used to annotate the sequences. Prodigal (v2.6.3) software was used to predict coding DNA sequence, Hmmer v3.1b2 was used to predict rRNA and Aragorn v 1.2.38 was used to predict tRNA of the cp genome sequence and obtain the first annotation result. Blast (version: BLAST 2.+, E-value ≤1^e-5^) software was used to compare the assembled sequence with its closely related species (*H*. *bogdanii*, NC 043839.1) to remove nuclear genome sequences and obtain the second annotation result. The final cp genome gene annotation was obtained after manual correction. The circular genome map of *H*. *brevisubulatum* was drawn using the OGDRAW v1.2 program [23]. The SSRs were analyzed using the Perl script MISA V1.0, and the minimum repeats of mono-, di-, tri-, tetra-, penta- and hexanucleotide were set to 8, 5, 3, 3, 3 and 3, respectively [22]. Vmatch v2.3.0 software identified dispersed repeats including forward, reverse, complement, and palindromic match repeats with a minimal length of 30bp, and hamming distance of 3 [11, 24, 25]. Tandem repeats were identified using Tandem Repeats Finder v. 4.09 [26]. Nucleotide A, T, C and G content at the third position of synonymous codons was acquired via the program Codon W (version 1.3, https://sourceforge.net/projects/codonw/). Parity rule 2 (PR2) analysis was employed to examine the nucleotide usage bias in the coding genes of *Hordeum species* [27].

### Phylogenetic analysis

Thirty-five species, including 5 species from *Hordeum*, were used for phylogenetic analysis. The cp genomes of another 34 species in fasta format were downloaded from NCBI database. Multiple sequence alignment was done using MAFFT software, which was trimmed by trimAl (v1.4.rev15), and then the (GTR)+G model was used in RaxML (v 8.2.10) software, as suggested, by 1000 bootstrap replicates with the default tree search algorithm of hill-climbing. The phylogenetic tree was constructed with the corresponding GeneBank number labeled near each species.

### Genome structure comparison

Based on the above results of the phylogenetic analysis, the complete cp genomes of five *Hordeum* species were compared using the mVista program with the shuffle-LAGAN mode using the annotation of *H*. *brevisubulatum* as reference [24]. The IRscope tool [28] was used to visualize the genes on the boundaries of the junction sites of the five *Hordeum* cp genomes.

## Results

### Features of *H*. *brevisubulatum* Cp genome

**Fig 1:**
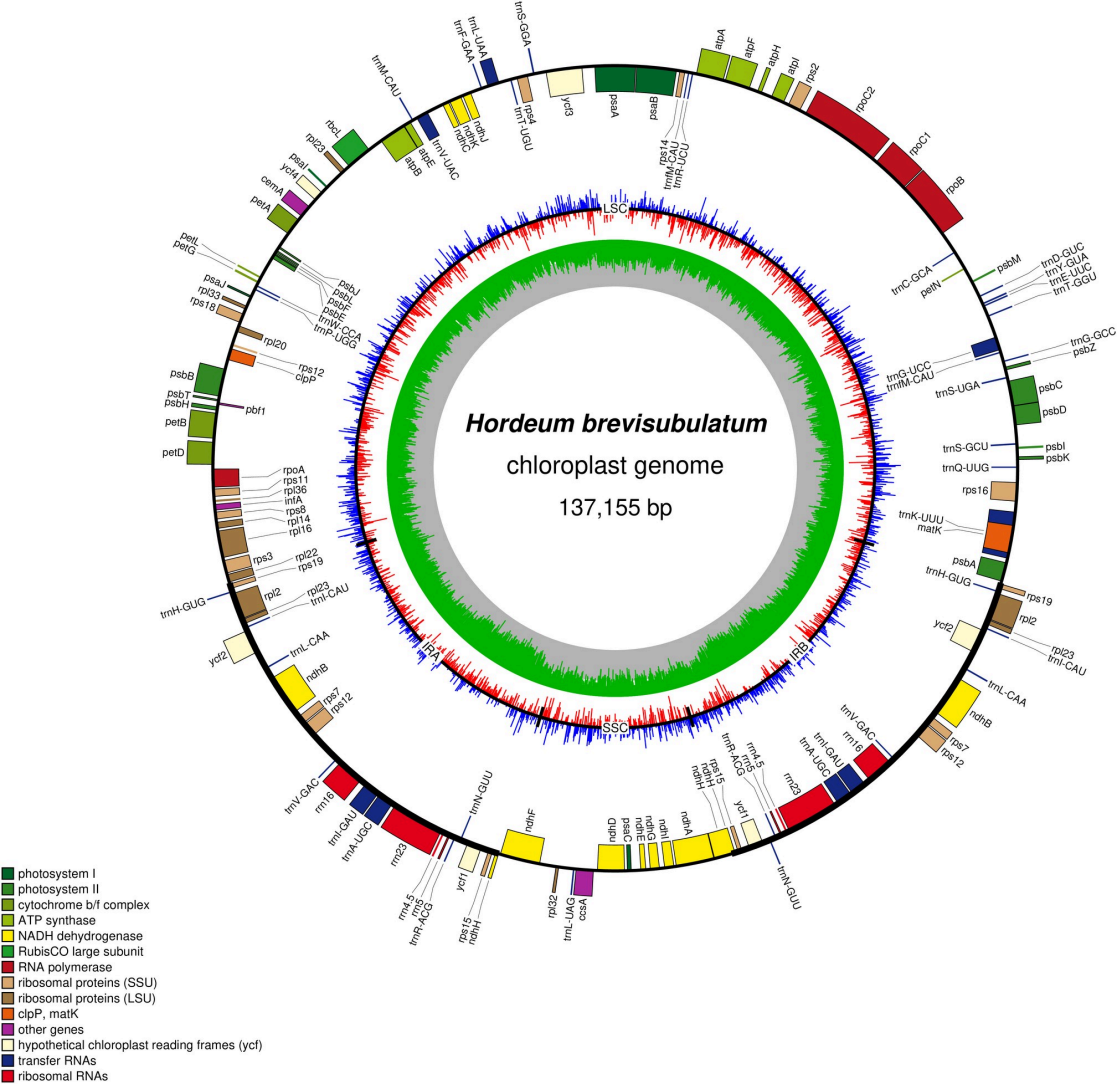
Cp genome map of *H*. *brevisubulatum*. Genes inside the circle are transcribed clockwise, and those outside are transcribed counter-clockwise. Genes of different functions are color-coded. The darker gray in the inner circle shows the GC content, while the lighter gray shows the AT content. The red and blue lines indicate GC skew, the red means GC skew greater than zero while the blue means GC skew smaller than zero.

The complete cp genome of *H*. *brevisubulatum* was 137,155 bp in length with a typical quadripartite structure, containing a pair of IR regions (43,174 bp), a LSC region of 81,175 bp, and a SSC region of 12,806 bp (Fig 1). The nucleotide composition of the *H*. *brevisubulatum* cp genome was biased towards A and T. All regions had different AT contents but all were AT rich. The AT content of the LSC, SSC, and IR regions and the whole cp genome were 63.80%, 67.91%, 56.14%, and 61.77%, respectively. It was obvious that the AT content of the IR region was lower than that of the LSC and SSC regions (Table 1). Base composition asymmetry (AT, CG) was found in a single strand of the *H*. *brevisubulatum* cp genome, except for in the IR region (Table 1).

**Table 1:** Base composition in a single strand of the *H*. *brevisubulatum* cp genome.

Region	A (%)	T (U) (%)	C (%)	G (%)	A+T (%)	G+C (%)
LSC	31.74	32.06	17.92	18.28	63.8	36.2
SSC	36.2	31.71	16.58	15.51	67.91	32.09
IR	28.07	28.07	21.93	21.93	56.14	43.86
Total	31	30.77	19.06	19.17	61.77	38.23

**Fig 2:**
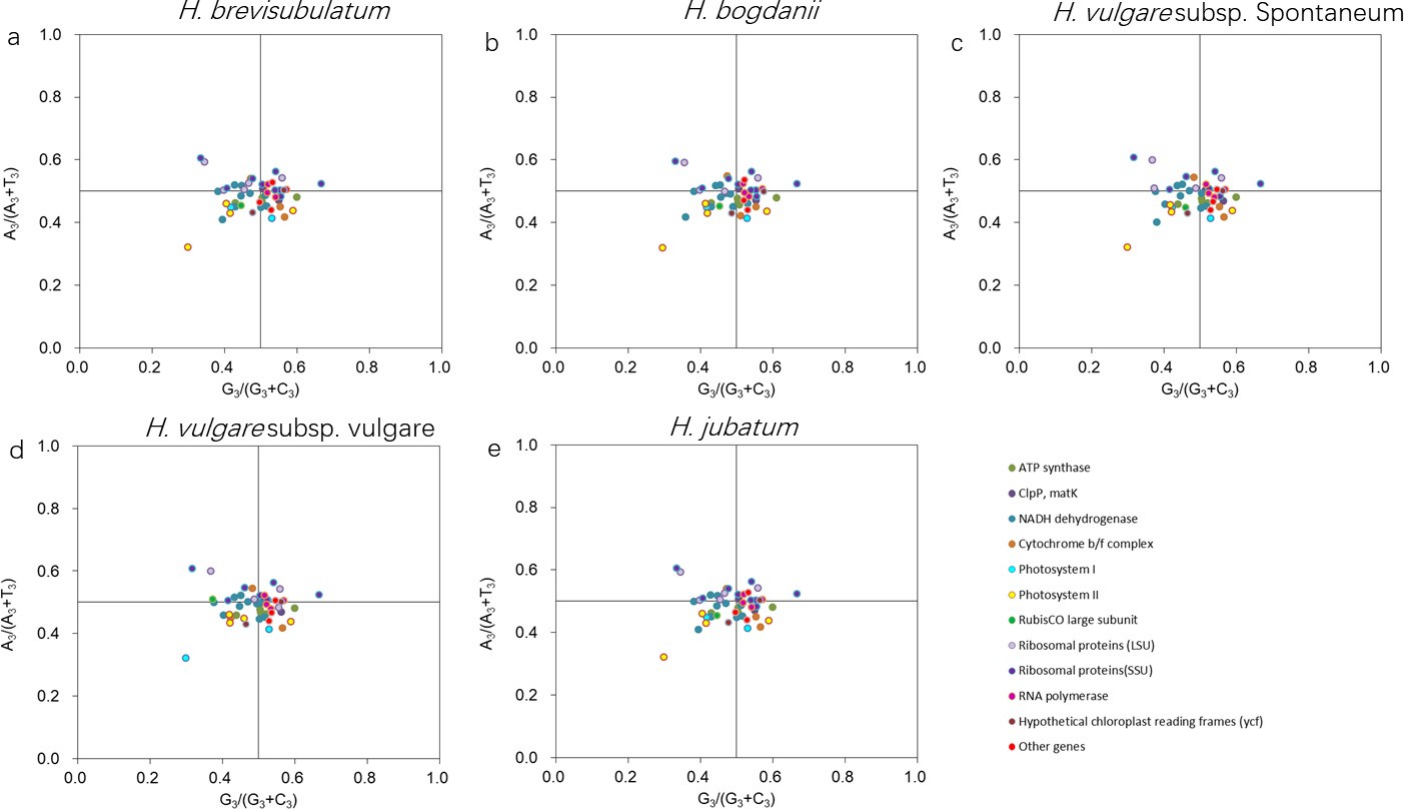
PR2 analysis for genes in the cp genomes of five *Hordeum* species. A_3_, T_3_, C_3_ and G_3_ represent nucleotide A, T, C and G content at the third position of synonymous codons, respectively.

Fifty, fifty, fifty-one and fifty-one Coding sequences (CDSs) with lengths larger than 300 bp were screened from the cp genomes of *H*. *bogdanii*, *H*. *vulgare* subsp. *vulgare*, *H*. *vulgare* subsp. *spontaneum* and *H*. *jubatum*, respectively. Together with 51 CDSs of *H*. *brevisubulatum*, they were used to carry out PR2 plot mapping analysis which was constructed to show the relationship between the values A_3_/(A_3_ + T_3_) and G_3_/(G_3_ + C_3_), and the data were distributed into four quadrants in a scatter diagram (Fig 2A–2E). It could be seen that along the ordinate, all five *Hordeum* species presented similar distributions with a majority of genes located in the third and fourth quadrants (in which the ratio of A_3_/(A_3_ + T_3_) < 0.5). However, along the abscissa, there were two types of distributions. A slightly larger number of genes of *H*. *brevisubulatum* (Fig 2A), *H*. *vulgare* subsp. *vulgare* (Fig 2B), *H*. *vulgare* subsp. *spontaneum* (Fig 2C) and *H*. *jubatum* (Fig 2D) were distributed on the G<C side than on the G>C side, while an equal amount genes of *H*. *bogdanii* were distributed on both sides (Fig 2E).

In the cp genome of *H*. *brevisubulatum*, a total of 130 functional genes were annotated including 83 protein-coding genes, 39 tRNA genes, 8 rRNA genes. All of these genes were classified into four categories (S1 Table). The protein-coding genes, tRNA genes, and rRNA genes accounted for 63.85%, 30.00%, and 6.15% of all of the annotated genes, respectively. Among the annotated genes, 22 genes were duplicated in the IR regions, including 4 rRNA genes (*rrn4*.*5*, *rrn5*, *rrn16*, and *rrn23*), 10 protein coding genes (*ndhB*, *ndhH*, *rpl2*, *rpl23*, *rps15*, *rps12*, *rps19*, *rps7*, *ycf2* and *ycf3*), and 8 tRNA genes.

In the *H*. *brevisubulatum* cp genome, 16 different genes harbored one intron, including 6 tRNA genes (*trnK-UUU*, *trnG-UCC*, *trnL-UAA*, *trnV-UAC*, *trnI-GAU*, and *trnA-UGC*) and 10 protein coding genes, whereas the protein coding genes of *ycf3 and rps12* contained two introns (S1 Table).

### Repeat sequence and SSRs analysis

Five categories of repeat sequences were detected and analyzed in the five *Hordeum* cp genomes (Table 2). The number of repeats was highest in *H*. *jubatum* (147) and lowest in *H*. *vulgare* subsp. *vulgare* (126). Tandem, palindromic and forward were the most common repeat sequence type. Complement and reverse repeats were found only in *H*. *jubatum*. In five *Hordeum* species, majority of the tandem repeats were less than 10 bp, more than half forward repeats were between 30–35 bp, and up to a half of palindromic repeats were between 30–35 bp (Table 2).

**Table 2:** Summary of repeat sequences and SSRs in five *Hordeum* chloroplast genomes.

Species	*Hordeum brevisubulatum*	*Hordeum vulgare* subsp. *vulgare*	*Hordeum jubatum*	*Hordeum vulgare* subsp. *spontaneum*	*Hordeum bogdanii*
Total dispersed repeat	53	47	70	47	45
Complement	0	0	2	0	0
Forward	34	30	43	31	29
30-35bp	21	19	22	19	19
36-45bp	7	8	10	9	7
46-65bp	3	0	4	0	2
66-100bp	1	1	4	1	0
>100bp	2	2	3	2	1
Palindromic	19	17	23	16	16
30-35bp	8	8	9	8	8
36-45bp	4	4	4	4	4
46-65bp	3	0	4	0	2
66-100bp	1	1	2	1	0
>100bp	3	4	4	3	2
Reverse	0	0	2	0	0
Total tandem repeat	85	79	77	80	84
<10bp	61	64	59	64	61
10-20bp	19	13	12	14	18
21-30bp	5	2	6	2	5
Total SSR number	182	181	187	180	182
mononucleotide	120	122	128	122	119
dinucleotide	6	6	6	6	7
trinucleotide	45	42	42	41	45
tetranucleotide	10	10	10	10	10
hexanucleotide	1	1	1	1	1

Except for the IR regions, a total of 52 dispersed repeats were found in *H*. *brevisubulatum* cp genome, including 34 forward and 18 palindromic repeats, with no reversed and complement repeats detected (S2 Table). In identified repeats, 35 repeats were 30–39 bp in length, 7 repeats were 40–49 bp, 10 repeats were longer than 50 bp and the longest repeat was 157 bp (S2 Table). As shown in S2 Table, a total of 31, 2, 2, and 2 repeats were detected in the LSC, SSC, IRa, and IRb regions, respectively. Twelve repeats were located in both the LSC and IRa/IRb regions and 3 repeats were located in both the IRa and IRb regions. The majority of repeat sequences were located in the intergenic spaces (IGS) (37%) and gene coding regions (38%, namely *ropC2*, *rps18*, *psaA*, *psaB*, *infA*, *rps3*, and *psbM*), while a minority of the repeats were detected in the intron (13%, namely the intron of *rps12*, *ndhB* and *ycf3*). It was worth noting that the *ropC2* gene possessed the largest number of repeats.

**Fig 3:**
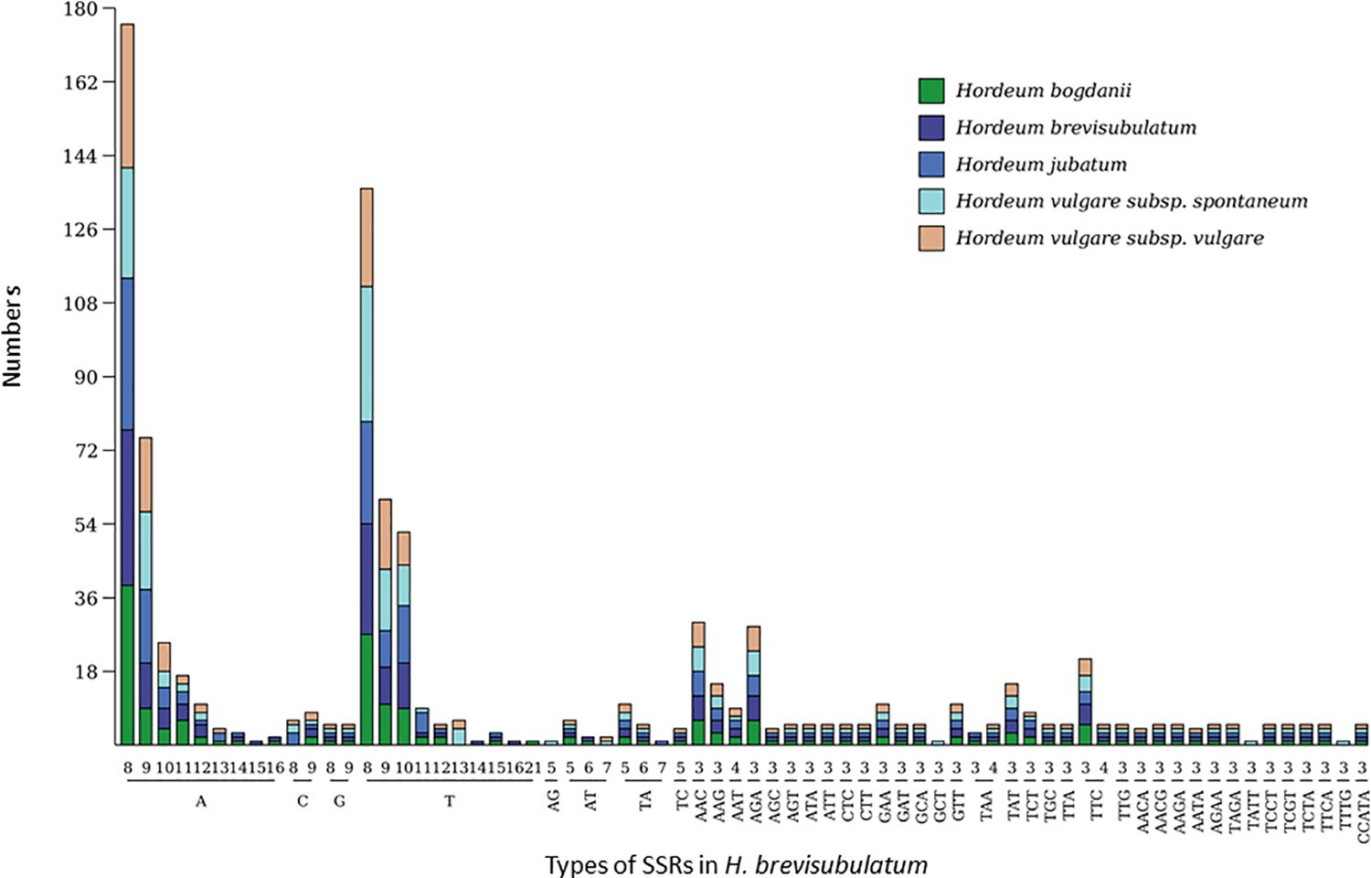
Statistical information of SSRs in *H*. *brevisubulatum*.

A total of 182 SSRs were identified in the *H*. *brevisubulatum* cp genome using MISA perl script (S3 Table). Among these SSRs, 58 types of repeat units were found, namely, 19 mononucleotides, 5 dinucleotides, 23 trinucleotides, 9 tetranucleotides, and 1 pentanucleotide (CCATA) (S3 Table). Of the 182 total SSRs, 120 SSRs were mononuclotides, accounting for 66%, and only 4 (2 of type C9 and 1 each of type G8 and G9) did not contain A or T (Table 2). Moreover, oligo A and oligo T made up 29% and 35% of the total 182 SSRs, respectively in the *H*. *brevisubulatum* cp genome. The SSR varied in number and type depending on the species. *H*. *jubatum* had the most SSRs (187) and *H*. *vulgare* subsp. *spontaneum* had the least (180) (Table 2). *H*. *brevisubulatum* had the most types (58) and *H*. *vulgare* subsp. *spontaneum* had the least (52) types of repeat units. A15, T14 and T16 only existed in *H*. *brevisubulatum*, T21 only existed in *H*. *bogdanii*, TA7 only existed in *H*. *jubatum*, while GCT3, TATT3 and TTTG3 only existed in *H*. *vulgare* subsp. *spontaneum* (Fig 3).

### Phylogenetic analysis

**Fig 4:**
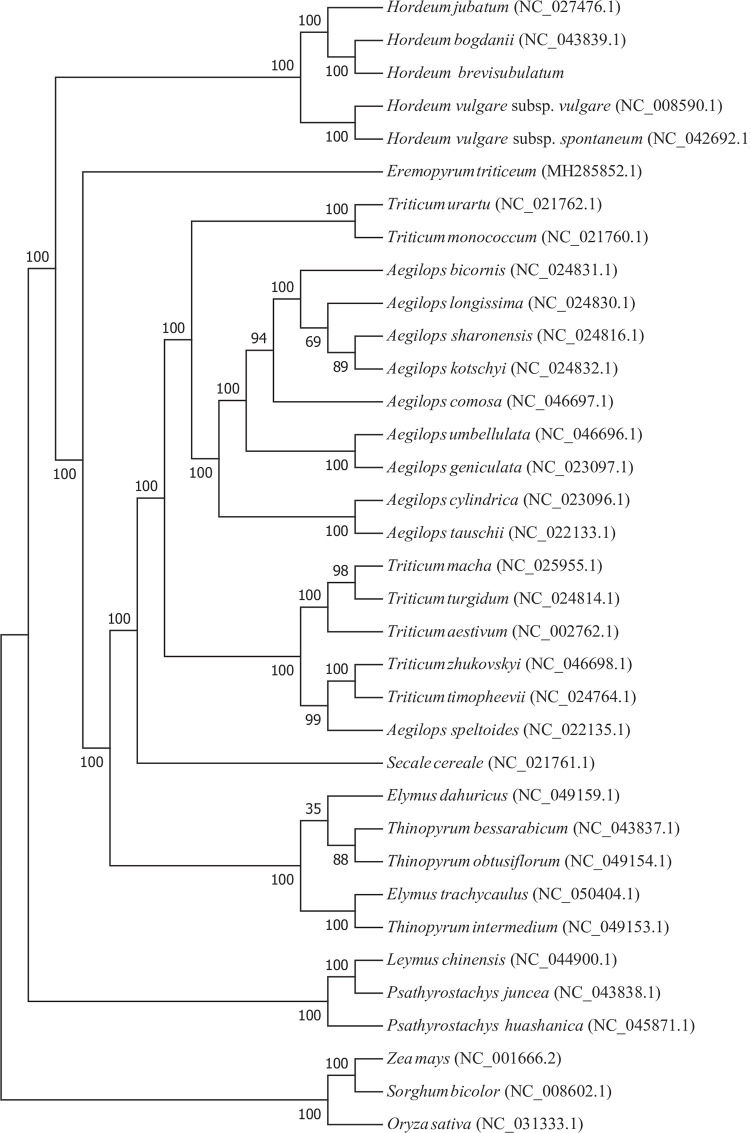
Phylogenetic relationships of 35 species based on complete cp genomes. The bootstrap values were based on 1000 replicates and are shown next to the branches. The cp genome accession numbers from GenBank are labeled near the names of plant species.

In the current work, the published cp genomes of 31 species from Triticeae, Gramineae family, namely 10 *Aegilops* species, 2 *Elymus* species, 1 *Eremopyrum* species, 4 *Hordeum* species, 1 *Leymus* species, 2 *Psathyrostachys* species, 1 *Secale* species, 3 *Thinopyrum* species, and 7 *Triticum* species, were downloaded from NCBI to explore the phylogenetic relationship with *H*. *brevisubulatum*. In addition, 3 important crops belonging to the Gramineae family, including *Zea mays* from Maydeae, *Sorghum bicolor* from Andropogoneae, and *Oryza sativa* from Oryzeae were used as outgroups to construct the evolutionary phylogenetic tree. The results showed that *Hordeum* species had a closer relationship with species from *Aegilops*, *Triticum*, *Secale*, *Thinopyrum*, *Elymus*, *Thinopyrum*, and *Eremopyrum* than with species from *Psathyrostachys* and *Leymus* in Triticeae (Fig 4). *H*. *jubatum*, *H*. *brevisubulatum*, *H*. *bogdanii*, *H*. *vulgare* subsp. *vulgare*, and *H*. *vulgare* subsp. *spontaneum* clustered into a big group with much higher internal resolution. Moreover, as shown in Fig 4, all 5 *Hordeum* species were divided into two subgroups. *H*. *vulgare* subsp. *vulgare* was close to *H*. *vulgare* subsp. *spontaneum*, and separated from *H*. *jubatum*, *H*. *brevisubulatum* and *H*. *bogdanii*. In the subgroup, *H*. *brevisubulatum* clustered closer to *H*. *bogdanii* than to *H*. *jubatum*.

### Comparative analysis of genome structure

The complete cp genome sequence of *H*. *brevisubulatum* was compared to four other *Hordeum* species (Table 3). *H*. *brevisubulatum* had the largest cp genome size and the largest LSC and SSC region, while *H*. *vulgare* subsp. *vulgare* had the smallest cp genome, smallest LSC, and the most annotated genes. The LSC length of *H*. *brevisubulatum* was 12,806 bp, which was 105 bp, 140 bp, 28 bp and 24 bp longer than *H*. *vulgare* subsp *vulgare*, *H*. *jubatum*, *H*. *vulgare* subsp. *spontaneum*, and *H*. *bogdanii*, respectively. The IR regions of *H*. *brevisubulatum*, *H*. *bogdanii*, *H*. *vulgare* subsp. *vulgare* and *H*. *vulgare* subsp. *spontaneum* were nearly the same length, but *H*. *jubatum* was a little shorter. As a domesticated barley variety, *H*. *vulgare* subsp. *vulgare* had the smallest genome size but encoded the most genes, 8–10 genes more than other four species, which were mainly tRNA genes. The GC content of the cp genomes of five *Hordeum* species differed slightly in four regions, with the highest content in the IR region (43.81–43.87%).

**Table 3:** Summary of five *Hordeum* chloroplast genome features.

Species	*Hordeum brevisubulatum*	*Hordeum vulgare* subsp. *vulgare*	*Hordeum jubatum*	*Hordeum vulgare* subsp. *spontaneum*	*Hordeum bogdanii*
Accesion	.	NC_008590.1	NC_027476.1	NC_042692.1	NC_043839.1
Genome size(bp)	137155	136462	136826	136536	136968
LSC length(bp)	81175	80597	80901	80612	81047
SSC length(bp)	12806	12701	12665	12778	12747
IR length(bp)	21587	21582	21630	21573	21587
Number of genes	130	139	129	130	131
Number of Protein-coding genes	83	83	82	83	83
Number of tRNA genes	39	48	39	39	40
Number of rRNA genes	8	8	8	8	8
All GC content(%)	38.23	38.32	38.24	38.30	38.24
LSC GC content(%)	36.20	36.31	36.19	36.30	36.20
SSC GC content(%)	32.09	32.33	32.32	32.25	32.12
IR GC content(%)	43.86	43.83	43.81	43.84	43.87

**Fig 5:**
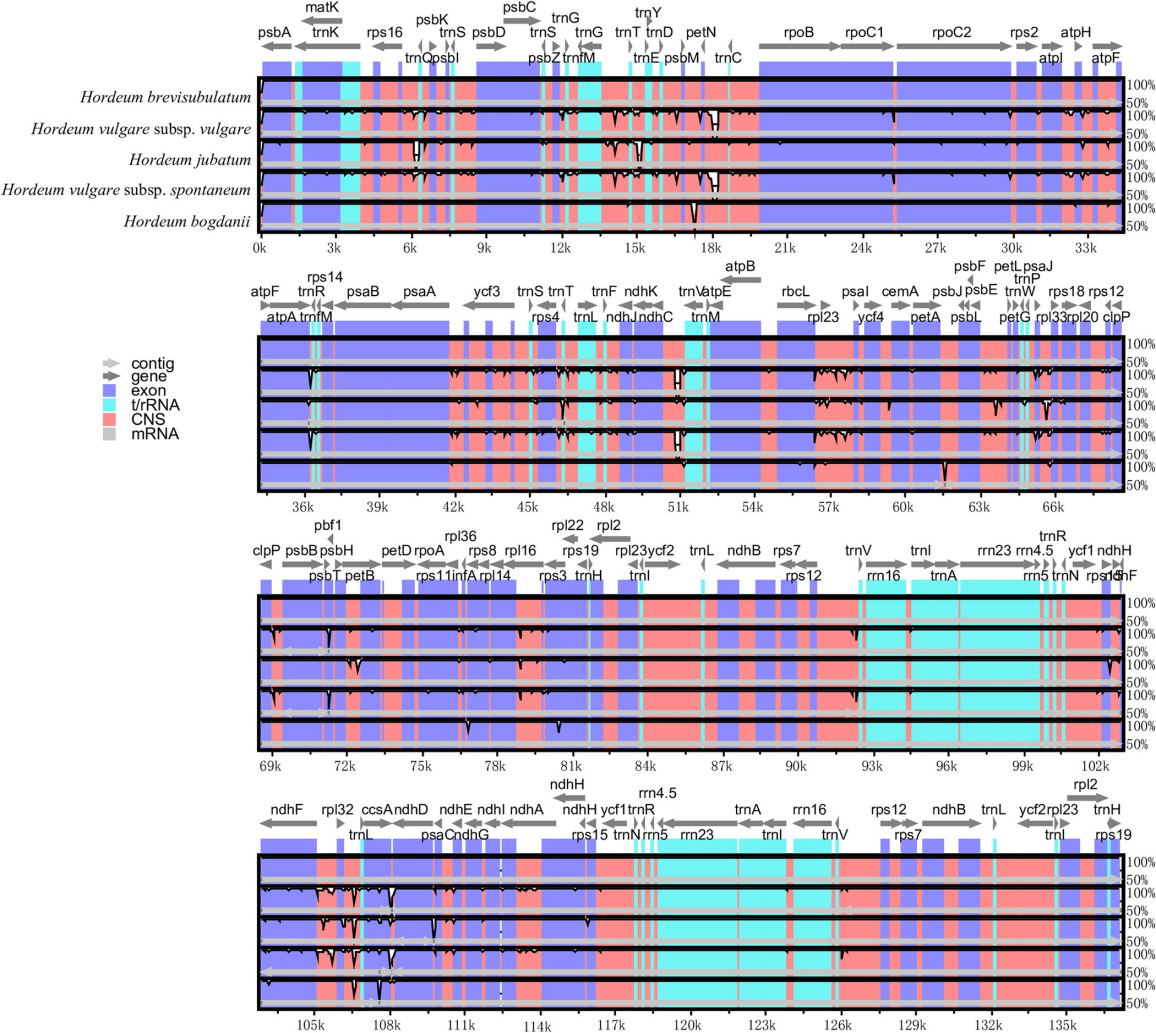
Complete cp genome comparison of five species using the cp genome of *H*. *brevisubulatum* as a reference. The grey arrows and thick black lines above the alignment indicate the gene’s orientations. The Y-axis represents identity ranging from 50% to 100%. UTR, untranslated regions. CNS, conserved non-coding sequences.

To further present cp genome divergence among the five *Hordeum* species, sequence identity was compared using mVISTA with *H*. *brevisubulatum* as a reference (Fig 5). Generally, the LSC and SSC regions were more divergent than IR regions and noncoding regions were more divergent than coding regions. Consistent with the results of phylogeny analysis, the cp genomes of these five *Hordeum* species could be divided into three groups. *H*. *brevisubulatum* was analogical to *H*. *bogdanii*, and *H*. *vulgare* subsp. *vulgare* was analogical to *H*. *vulgare* subsp. *spontaneum*. They were all different from *H*. *jubatum*. Among the five *Hordeum* species, prominent divergence occurred in the IGS of *trnG-GCC*-*trnY-GUA*, *psbM*-*petN*, *petN*-*trnC-GCA*, *petA*-*psbI*, *ndhC*-*trnV-UAC*, *rbcL*-*pasI*, and *rpl23*-*ndhD* as well as coding regions of *ccsA*, which could be used as reliable markers for phylogenetic studies. Between *H*. *brevisubulatum* and *H*. *bogdanii*, there were significant differences in the IGS of *psbM*-*petN*, *petA*-*psbL*, *rpl32*-*trnL-UAG*, and *ccsA*-*ndhD*, as well as the coding regions of *infA*, *rps3*, and *ccsA*.

**Fig 6:**
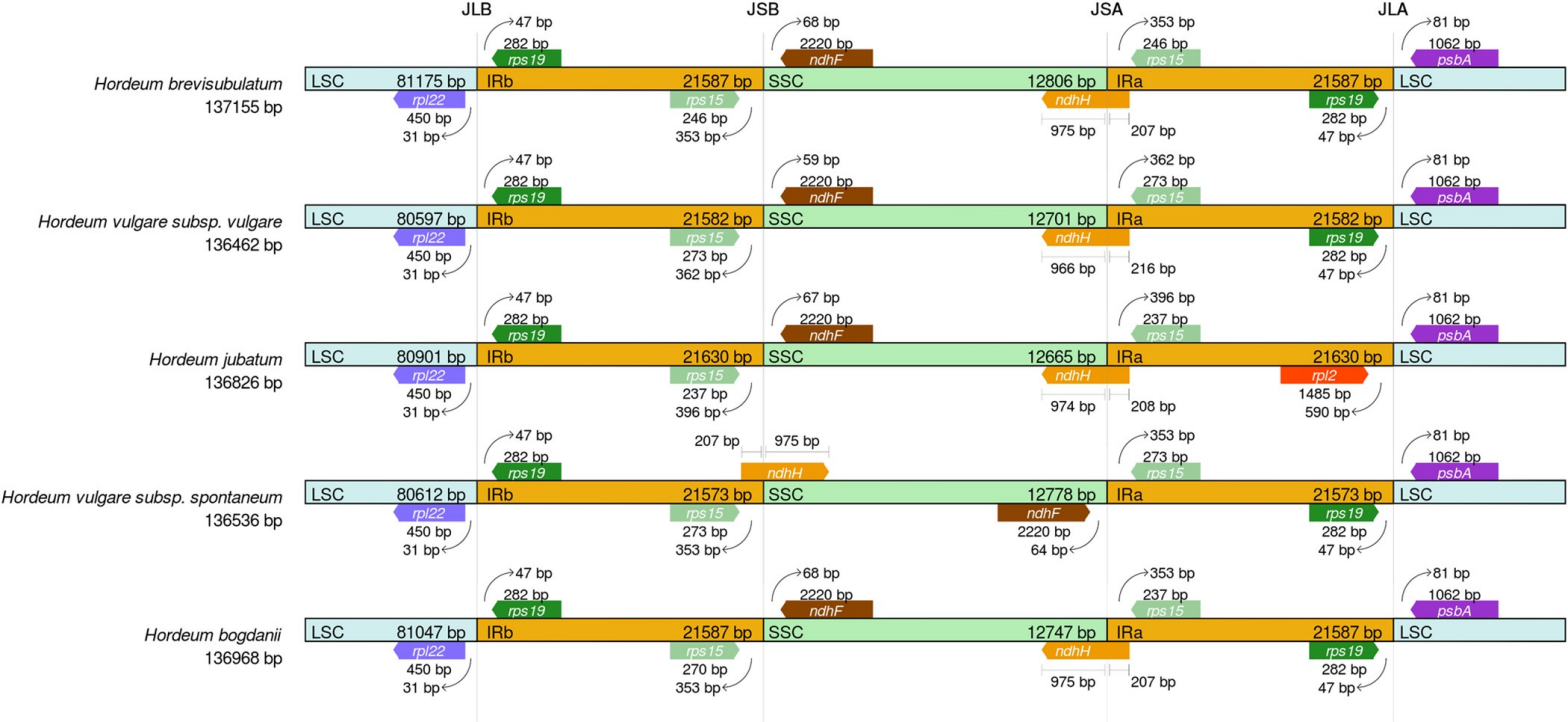
Comparison of the large single copy (LSC), small single copy (SSC), and inverted repeat (IR) regions in cp genomes of five *Hordeum* species. Genes and regions are denoted by colored boxes. The gaps between the genes and the boundaries are indicated by the base lengths (bp). Extensions of the genes are indicated above the boxes. The lengths of the four regions are also shown in the box.

Detailed comparisons of the IR boundaries among the five *Hordeum* species are presented in Fig 6. Seven different genes were located at the juncture of the LSC/IRb (*rps19*, and *rpl22*), IRb/SSC (*ndhF*, rps15 and *ndhH*), SSC/IRa (*ndhH*, *ndhF*, and *rps15*), and IRa/LSC borders (*rps19*, and *psbA*). The cp genomes of these five *Hordeum* species were relatively conserved except for two major divergences—at the IRa/LSC border in *H*. *jubatum* and at the SSC/IRa border in *H*. *vulgare* subsp. *spontaneum*. In the cp genomes *of H*. *brevisubulatum*, *H*. *vulgare* subsp. *vulgare*, *H*. *jubatum* and *H*. *bogdanii*, the *ndhH* gene crossed the SSC/IRa border with the larger part located in the SSC region and the *ndhF* gene located at the IRb/SSC border. In *H*. *vulgare* subsp. *spontaneum*,. the *ndhH* gene crossed the IRb/SSC border with 975 bp located in SSC region and the *ndhF* gene near the SSC/IRa boder. Different from other four species which possessed *rps19* in the SSC region with 47 bp away from the IRa/LSC border, there was no *rps19* gene near the IRa/LSC border in *H*. *jubatum*.

## Discussion

The cp genome is very useful in identifying closely related, breeding-compatible plant species. In this study, the relationship between 5 *Hordeum* species and among 35 species in Triticeae were examined. This could be beneficial for traditional variety breeding in *H*. *brevisubulatum* since it is mainly breeding cultivated crops with their wild relatives. Furthermore, the publication of the cp genome of *H*. *brevisubulatum* will enable researchers to build transgene cassettes for further cp genome engineering since it could provide both flanking and regulatory sequences. Furthermore, it would enable the construction of species-specific chloroplast vectors. There were many successful examples of the use of cp genome engineering to improve abiotic resistance. For example, salt tolerance in carrots was enhanced by over-expressing the *badh* gene in its cp genome [29] and tabacco salt resistance was improved by the expression of γ-TMT in its chloroplast [30]. However, some factors, including the need to identify species-specific vectors, difficulties in homogenization, and the need to examine the rule of cp protein expression have hindered cp genome engineering development [10].

### Cp genome feature of *H*. *brevisubulatum*

The cp genomes of terrestrial plants are not only conserved in structure but also in gene and intron content. Generally, the cp genome is a typical quadripartite structure, except for IR segment divergence. For example, the cp genome of alfalfa comprises only one copy of the IR region [31], whereas in some algae and conifers, there are no IR regions [11, 12]. With the loss of IR regions or entire gene families, linear cp genomes have been found [32]. The cp genomes of the earliest diverging angiosperms harbor 113 different genes, including 16 duplicated in IR regions, for a total of 129 genes. These genes play vital roles in photosynthesis, self-replication, and biosynthesis related to amino acids, fatty acids, pigments, starch, and other unknown functions. However, the loss of genes has been observed in many angiosperms [14]. Haberhausen and Zetsche [33] found the loss of the *ndh* genes in *Cuscuta reflexa*. Millen et al. [34] found the loss of the *infA* gene in *Lemna minor*, and proposed that these genes had been transferred into the nucleus or did not participate in critical life development. In this study, the cp genome of *H*. *brevisubulatum* was a typical quadripartite structure that harbored 108 different genes with 22 genes duplicated in the IR regions, verifying highly conserved traits of cp genomes in gene content and genome organization. Tang et al. [35] compared gene sequences of *accD*, *ycf1*, and *ycf2* in *Typha latifolia* with 13 other Gramineae species and found that only conserved segments of these genes were retained in these 13 species. This suggested that sequences of *accD*, *ycf1*, and *ycf2* genes were gradually eliminated during the evolution of Gramineae [35]. The *accD* gene was lost in all five *Hordeum* species, while only segments of *ycf1* and *ycf2* occurred in *H*. *brevisubulatum* and was lost in *H*. *vulgare* subsp. *vulgare*, *H*. *jubatum*, and *H*. *bogdanii*, suggesting gene degradation during evolution.

Among the 129 total genes in the earliest diverging angiosperms, 22 genes contain introns. Intron absence within protein-coding genes usually occurs in monocot and eudicot clades, such as *Cicer arietinum*, *Manihot esculenta*, and *H*. *vulgare* [10, 14, 35]. In the *H*. *brevisubulatum* cp genome, a total of 17 different genes contained introns. Compared with the earliest diverging angiosperms, introns of four genes, namely, *clpP*, *rpoC1*, *rpoC2*, and *ycf1* were absent. Due to the intron absence of *clpP*, which formerly harbored two introns, only the *ycf3* gene harbored two introns. This was different from *Fagopyrum dibotrys*, *Quercus acutissima*, and *Sinapis alba*, in which both *ycf3* and *clpP* contained two introns [36–38]. Intron absence in *clpP* extensively occurred in Onagraceae, Oleaceae, and gymnosperms [14], as well as in Gramineae, except for *Anomochloa marantoidea* [33]. The genes with intron absence might endow *H*. *brevisubulatum* with diverse functions in protease, RNA polymerase and ribosomal proteins.

AT was abundant in the *H*. *brevisubulatum* cp genome. This was consistent with findings in *P*. *mume*, *S*. *alba*, *Q*. *acutissima*, *P*. *pretense*, *D*. *grandiflorum*, and *Dicliptera* species [11, 26, 38–41]. Due to the bias of the replication mechanism, gene orientation and transcribed/non-transcribed strands, as well as cytosine deamination, strand composition asymmetry were universally existed in prokaryotic and eukaryotic chromosomes [42–44]. The asymmetry between A and T and between C and G in a single strand were evident in the LSC, SSC, and cp genome of *H*. *brevisubulatum*. Moreover, PR2 analysis further verified that a majority of genes had a bias toward T over A in the coding strand in all five *Hordeum* species, and a slight bias toward C over G in the coding strand in four other *Hordeum* species, expect for *H*. *bogdanii*. A direct cause of base composition asymmetry was replication mechanism, and nevertheless that asymmetries between the coding and noncoding strands, the cause mechanisms such as transcription-induced mutation or codon choice, are responsible for a large part of the nucleotide skews [45]. Since the leading strand is synthesized continuously, whereas as the lagging strand is replicated in a fragmented manner, the 2 DNA strands can be highly asymmetric in nucleotide composition. Moreover, the deamination events in the nontranscribed strand contribute to C→T substitutions, which accumulates such changes at a two-to threefold higher rate than the complementary transcribed strand [44, 45]. Nevertheless, the impact of the replication mechanism on base bias differed for GC and AT asymmetries. The effect of replication on the GC skew was generally very strong, and the AT skew was exclusively caused by coding sequence-related mechanisms [45–47]. The reasons that lead to the base asymmetry in *H*. *brevisubulatum* cp genome need to be studied in depth.

### Molecular markers

Repeat sequences are found extensively in plant cp genomes [48]. Repeat sequences differ in type, number, and location depending on species. In four species of the *Dicliptera genus* and *Nasturtium officinale* [24, 26], three types–forward, palindromic, and reversed repeats were found. In the *Dicliptera peruviana* cp genome, there were 56 dispersed repeats, and in *Dicliptera montana*, there were 41 dispersed repeats. Among the five *Hordeum* species, *H*. *jubatum* possessed the most of dispersed repeats while *H*. *bogdanii* had the least. Moreover, *H*. *jubatum* had four types of dispersed repeats, and the other four species only had two types. The most tandem repeats were in *H*. *brevisubulatum*, and 52 dispersed repeat sequences were detected from the categories of forward and palindromic. The majority of identified dispersed repeats in the *H*. *brevisubulatum* cp genome were located in the LSC region, showing an uneven distribution. Moreover, 75% were concentrated in IGS, which are highly variable [26] and gene coding regions. In *N*. *officinale*, most were located in IGS and introns. Repeat sequences could be used as indicators of mutational hotspots [49]. In this study, the *rpoC2* gene possessed the largest number of repeats, and similar phylogeny relationships were obtained when using only this gene to construct a new phylogeny tree. Hence, we reasoned it might be used as a potential molecular marker for further studies in *Hordeum* species.

SSRs, also termed microsatellites, are rich in cp genomes [24, 26, 36, 50, 51] and composed of one or a few consecutive repeated nucleotides. Since genetic information loaded on the cp genome is only inherited from the maternal line, SSRs in cp genomes are sensitive to population genetic effects [52] and have been used widely to trace the maternal gene flow in populations and in research in population evolution and polymorphism [49]. SSRs exhibit variations in number and type according to species. In five *Hordeum* species, only one pentanucleotide (CCATA) SSR was found, while two pentanucleotide and two hexanucleotide SSRs were found in *S*. *alba* [36] and only mononucleotide, dinucleotides, trinucleotides, and tetranucleotides were detected in *Dicliptera species* [26], suggesting genetic diversity among different species [36]. In the cp genome of *H*. *brevisubulatum*, oligo adenine and oligo T repeats accounted for 64% of the total SSRs. These results were consistent with the findings in *S*. *alba*, *N*. *officinale* and *Raphanus sativus* [24, 36, 49], verifying that oligo adenine and oligo T repeats were common features of cp genomes. Only 4 SSRs did not contain A or T nucleotide, providing further evidence for A and T nucleotide bias in *H*. *brevisubulatum*. Among the five *Hordeum* species, except for *H*. *vulgare* subsp. *vulgare*, there were unique SSRs for each species, which could be used as molecular markers to distinguish different species. Therefore, the analysis of repeat sequences and SSRs in *H*. *brevisubulatum* and the other four *Hordeum* species further illustrated its cp genome characteristics and provided strong evidence in identifying molecular markers for further study in mutational hotspots, phylogenetic studies, species delimitation, haplotype recognition, population genetic analysis, and crop breeding [24, 49–51, 53].

### Phylogenetic relationships and genome structure comparison

The cp genome is considered to be a perfect model for evolutionary research and has been used widely in studies in plants including in cereals, cottons, trees, vegetables, ornamental plants, medical plants, and forages [24, 26, 37–39]. A robust phylogenetic tree would help improve target plants and facilitate sustainable conservation strategies [10]. To examine the relationship between *H*. *brevisubulatum* and other species in Triticeae, a phylogenetic tree based on cp genomes was constructed among 35 species. The results demonstrated a significant relationship among five species of the *Hordeum* genus (Fig 4). *H*. *brevisubulatum* had a closer relationship with *H*. *bogdanii* than did *H*. *jubatum* and *H*. *vulgare* subsp. *vulgare* was close to *H*. *vulgare* subsp. *spontaneum*. This was in agreement with Bernhardt et al., who reported that *H*. *vulgare* was far from *H*. *jubatum* [54]. Although cp genomes are relatively conserved in gene composition and structure, important alterations occur due to gene or intron fragment loss, gene rearrangement, and expansion, extraction or loss of the IR region [35, 55, 56]. To fully demonstrate genome divergence among five closely related *Hordeum* species, including *H*. *brevisubulatum*, *H*. *bogdanii*, *H*. *vulgare* subsp. *vulgare*, *H*. *jubatum* and *H*. *vulgare* subsp. *spontaneum*, the genome structure was compared by analyzing genome features, sequence identity, and IR borders.

Low divergence among these five *Hordeum* species was found, revealing that the cp genomes were conserved. IR regions were more conserved than LSC and SSC regions and coding regions had less divergence than noncoding regions. This might be related to copy corrections during gene conversion [26] and has been reported in other angiosperms, such as in the *Prunus*, *Sinapis*, *Quercus*, *Dicliptera*, and *Nasturtium* genera [11, 24, 26, 36, 38]. The *ndhH* gene crossed the SSC/IRa border in *H*. *brevisubulatum*, *H*. *vulgare* subsp. *vulgare*, *H*. *jubatum*, and *H*. *bogdanii*. However, in *Quercus* species and *N*. *officinale*, the *ycf1* gene crossed the SSC/IRa region [24, 38] and in *Brassica juncea*, there was no gene that crossed the SSC/IRa border [36], suggesting genetic diversity in different species. The genome size variation of the five *Hordeum* species was due mainly to variation in IR regions and divergence at the SSC/IRa border (Table 3, Fig 6). The contraction or expansion of IR regions at the borders is widespread [41] and has been regarded as an evolutionary indicator that explains correlations among taxa [57, 58]. Significant changes in the gene location of *rps19* were found between *H*. *jubatum* and the other four species, due mainly to the contraction of the IR region after its expansion during evolution [35, 59]. Unlike the other four species, in *H*. *vulgare* subsp. *spontaneum*, the *ndhH* gene crossed the IRb/SSC border with a pseudo part at the SSC/IRa border. This kind of gene conversion during speciation was thought to occur from IR variations [26].

## Conclusions

In this study, the architecture of the *H*. *brevisubulatum* cp genome, including basic features, repeat sequence, SSRs, and phylogenetic relationships was described comprehensively. Then, the cp genome of *H*. *brevisubulatum* was compared with four other *Hordeum* species. The cp genome of *H*. *brevisubulatum* had a typical quadripartite structure and 130 functional genes were annotated. The gene of *accD* was lost in all five *Hordeum* species when compared with the earliest diverging angiosperms, and only segments of *ycf1* and *ycf2* were retained in *H*. *brevisubulatum* and absent in *H*. *vulgare* subsp. *vulgare*, *H*. *jubatum*, and *H*. *bogdanii*. Moreover, introns of *clpP*, *rpoC1*, *rpoC2*, and *ycf1* were absent in *H*. *brevisubulatum*. Base asymmetry were found in all five *Hordeum* species. In the coding strand, the bias of T over A existed in all five *Hordeum* species, while the bias of G over C differed. LSC and SSC regions were more divergent than IR regions, and IGS was more divergent than coding regions. Moreover, main divergence occurred at the SSC/IR borders in *Hordeum*. These findings, in combination with identified introns, repeat sequences, and SSRs enrich our knowledge on cp biology and genetic diversity of *H*. *brevisubulatum* and lay a strong foundation for further studies on molecular marker development, phylogenetic analysis, population studies and cp genome engineering.

## Supporting information

S1 Table List of genes annotated in the cp genome of *H*. *brevisubulatum*. Gene*: Gene with one intron; Gene**: Gene with two introns; #Gene: Pseudo gene; Gene (2): Number of copies of multi-copy genes.
(XLS)

S2 Table Repeat sequences in *H*. *brevisubulatum* cp genome. F, forward repeats; R, reversed repeats; IGS, intergenic spaces. Note: The first nucleotide position was located at the beginning of the LSC region, and the nucleotide position was counted along with the direction of LSC-IRa-SSC-IRb.
(XLS)

S3 Table Distribution of SSRs in the *H*. *brevisubulatum* cp genome. (XLS)

S1 File The full annotation of *H*. *brevisubulatum* cp genome. (TBL)

## Abbreviations

LSC: Large single copy
SSC: Small single copy
IR: Inverted repeat
Cp: Chloroplast
A: Adenine
T: Thymine
C: Cytosine
G: Guanine
IGS: Intergenic spaces
SSR: Simple sequence repeat
A_3_: Adenine content at the third position of synonymous codons
T_3_: Thymine content at the third position of synonymous codons
C_3_: Cytosine content at the third position of synonymous codons
G_3_: Guanine content at the third position of synonymous codons
